# Atypical Presentation of a Meningioma Masquerading as Trigeminal Neuralgia: A Diagnostic Challenge in the Emergency Department

**DOI:** 10.7759/cureus.117075

**Published:** 2026-09-28

**Authors:** Muntaha Naeem, Alisha S Mohammed

**Affiliations:** 1 Emergency Medicine, Basildon and Thurrock University Hospitals NHS Foundation Trust, Basildon, GBR; 2 Geriatrics, Cambridge University Hospitals NHS Foundation Trust, Cambridge, GBR

**Keywords:** atypical headache, cranial nerve compression, emergency medicine, meningioma, skull base imaging, sphenoid wing tumor, trigeminal neuralgia

## Abstract

A woman in her mid-40s presented to the Emergency Department (ED) following a sudden new-onset three-hour episode of numbness over the left maxilla and jaw, followed by electric shock-like facial pain triggered by talking and chewing. This occurred on a background of approximately four months of intermittent left-sided headache. Although the presentation initially suggested trigeminal neuralgia (TN) with concomitant pain, the prolonged sensory disturbance, left-sided proptosis, periorbital symptoms, and diplopia on left lateral gaze were clinically atypical and prompted further evaluation. Clinical examination demonstrated tenderness over the left ophthalmic (V1) and maxillary (V2) divisions of the trigeminal nerve, together with left-sided proptosis and diplopia on left lateral gaze. Neuroimaging was therefore undertaken. Computed tomography (CT) demonstrated an expansile sclerotic lesion involving the left greater wing of the sphenoid with a ground-glass appearance, initially raising suspicion of fibrous dysplasia. Subsequent magnetic resonance imaging (MRI) revealed a large *en plaque* meningioma involving the left sphenoid wing and middle cranial fossa, with orbital extension and compression of the left optic nerve, necessitating specialist neurosurgical evaluation. A separate right cerebellopontine angle meningioma was also identified. The patient underwent surgical resection of the symptomatic lesion, with histopathology confirming a World Health Organization (WHO) grade one meningioma. This case highlights the importance of thorough neurological assessment and early neuroimaging in patients presenting with atypical features of TN to exclude secondary causes of cranial nerve pathology more consistent with TN-like pain secondary to a structural lesion and enable timely management of any underlying lesions.

## Introduction

Trigeminal neuralgia (TN) is a disorder characterized by sudden, severe, episodic electric shock-like facial pain in the distribution of one or more branches of the trigeminal nerve [1,2]. Around 14-50% of patients report TN with concomitant pain, experiencing additional continuous pain in the same distribution as the sharp attacks. It is most often idiopathic but can less commonly also arise secondary to structural lesions such as tumors, vascular malformations, or multiple sclerosis in up to 10% of patients. Meningiomas arising from the sphenoid wing and skull base are rare but an important cause, as their proximity to the trigeminal nerve and its ophthalmic (V1) and maxillary (V2) divisions can result in compression or irritation of sensory fibers, producing facial pain and sensory disturbances that mimic primary TN [2,3]. This report discusses a case initially suspected to be primary TN but ultimately diagnosed as a large meningioma, illustrating the diagnostic challenges encountered in the Emergency Department (ED) when assessing atypical headache presentations with cranial nerve involvement.

## Case presentation

A 44-year-old woman presented to the ED with a background of intermittent left-sided temporo-occipital headaches persisting for around four months. These headaches were described as electric shock-like in nature, particularly in the temporal region, and worsened by actions such as coughing but not on lying down. The patient reported these headaches would occur every 2-3 days and start spontaneously, without preceding aura, and last a few hours before self-remitting without significant reported background pain. Additional reported features included mild photophobia, nausea without vomiting, and lacrimation during these episodes. However, the patient’s main concern for presenting was due to a recent three-hour episode of sudden-onset numbness over the left maxilla and jaw, followed by electric shock-type pain exacerbated by talking and chewing, which came on during long-distance driving. Associated symptoms included diffuse left facial pain, tenderness in the left temporal region, and periocular pain around the left eye. There was no history of tinnitus, vertigo or hearing changes, no vomiting or seizures, no systemic symptoms such as fevers, night sweats or weight loss, nor any paresthesia or weakness noted in either the upper or lower limbs.

The patient’s past medical history included sciatica and a previous breast fibroadenoma, and they were not on any regular medication. They were a non-drinker and ex-smoker with no history of recent trauma or systemic symptoms. There was no personal or family history of neurological disease or malignancy.

During the initial ED assessment, the patient was alert with a Glasgow Coma Scale score of 15/15, and observations showed the patient to be hemodynamically stable. On closer examination, the patient was noted to have left-sided proptosis and eyelid swelling. Cranial nerve examination revealed double vision on left lateral gaze, and the patient reported tenderness and subjective altered sensation affecting the left ophthalmic (V1) and maxillary (V2) distributions of the trigeminal nerve. Other cranial nerves were intact and there were no signs of dysphasia. Upper and lower limb neurological examination were normal, including both preserved power and sensation. Pupils were 3 mm bilaterally and both equal and reactive to light, with no relative afferent pupillary defect.

Subsequent formal ophthalmological assessment provided further characterization of the ocular findings. Visual acuity was 0.0 logMAR in both eyes, and color vision was preserved at 17/17 Ishihara plates bilaterally. Extraocular movements were full, although diplopia was elicited at extreme abduction of the left eye. Intraocular pressure was 17 mmHg in the right eye and 18 mmHg in the left eye. Hertel exophthalmometry measured 18 mm in the right eye and 23.5 mm in the left eye at a base of 118 mm, confirming approximately 5 mm of relative left-sided proptosis with significant periocular edema. The anterior segments were calm and corneas transparent, with corneal symptoms noted as a contributing factor to periocular pain. Both optic discs and maculae appeared healthy with no optic disc swelling. Overall, visual acuity and color vision remained preserved despite ongoing orbital involvement, with left-sided proptosis, periocular edema, periocular pain, and diplopia restricted to extreme positions of gaze.

The laboratory investigations were within normal limits (Table 1), with no biochemical evidence of significant systemic inflammation, hematological abnormality, electrolyte disturbance, or renal impairment.

**Table 1:** Laboratory investigations at presentation All laboratory parameters were within normal reference ranges, with no biochemical evidence of systemic inflammation, metabolic disturbance, or end-organ dysfunction. WBC: white blood cell count; Hb: hemoglobin; PLT: platelets; Na⁺: sodium; K⁺: potassium; CRP: C-reactive protein; ESR: erythrocyte sedimentation rate.

Investigation	Patient Value	Reference Range	Interpretation
White blood cell count (WBC), ×10⁹/L	7.4	4.0–11.0	Normal
Neutrophils, ×10⁹/L	3.37	2.0–7.5	Normal
Hemoglobin (Hb), g/dL	13.4	12.0–16.0	Normal
Platelets (PLT), ×10⁹/L	248	150–400	Normal
Sodium (Na⁺), mmol/L	138	135–145	Normal
Potassium (K⁺), mmol/L	4.1	3.5–5.0	Normal
Urea, mmol/L	7.5	2.5–7.8	Normal
Creatinine, µmol/L	75	45–90	Normal
Glucose, mmol/L	5.2	4.0–7.8	Normal
C-reactive protein (CRP), mg/L	1	<4	Normal
Erythrocyte sedimentation rate (ESR), mm/hr	4	<20	Normal

Given the history of transient unilateral facial numbness, proptosis, and cranial nerve involvement, a non-contrast CT head was performed. This revealed an expansile sclerotic lesion with ground-glass matrix in the left greater wing of the sphenoid bone, extending into the orbit and temporal bone, causing mass effect and proptosis (Figures 1A, 1B). The CT finding of a ground-glass matrix initially raised consideration of fibrous dysplasia; however, hyperostotic meningiomas may demonstrate similar appearances due to reactive bone formation and associated hyperostosis. This radiological overlap highlights the limitation of CT alone and underscores the importance of MRI for accurate lesion characterization and differentiation between these entities. 

**Figure 1:**
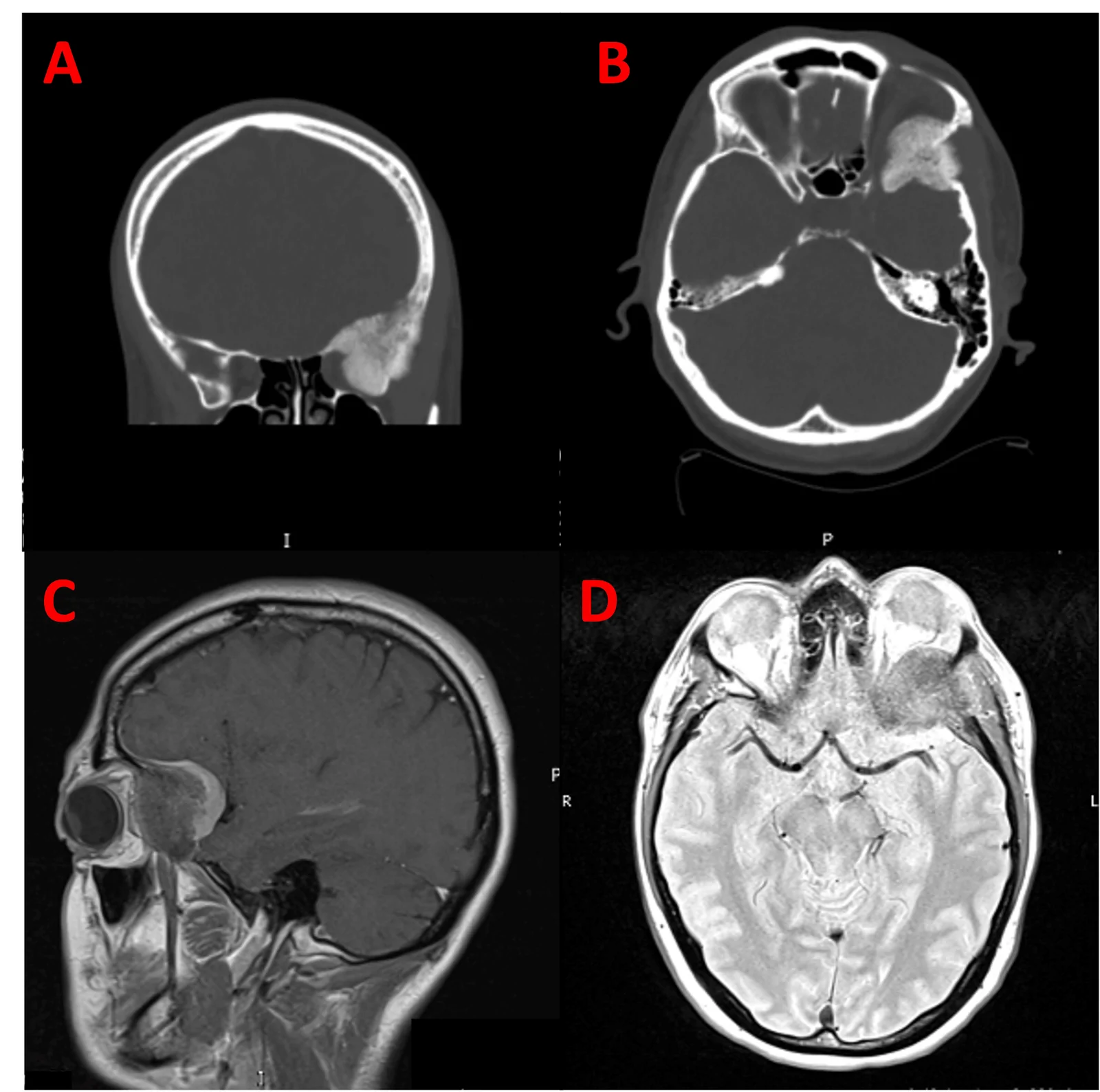
CT and MRI imaging demonstrating sphenoid wing meningioma with orbital involvement. Panel A: Coronal non-contrast CT head demonstrating an expansile, sclerotic mass involving the left greater wing of the sphenoid with a ground-glass matrix. There is extension into the left orbital roof and lateral wall, causing displacement of retro-orbital fat and proptosis of the left eye. Panel B: Axial non-contrast CT head showing the lesion encroaching on the left orbit and extending into adjacent frontal and temporal bones. The mass appears osseous with characteristic ground-glass appearance, raising differential considerations such as fibrous dysplasia or meningioma. Panel C: Sagittal MRI head illustrating a dural-based en plaque meningioma along the left sphenoid wing with hyperostosis and soft tissue components extending into the middle cranial fossa. Mass effect is evident on the left orbital structures and temporal lobe. Panel D: Axial post-contrast MRI head showing an enhancing lesion involving the left greater wing of the sphenoid with a dural tail, as well as compression of the left optic nerve at the orbital apex with associated proptosis. A separate right cerebellopontine angle meningioma mildly compressing adjacent brainstem structures was also reported.

The patient was admitted under the medical team for further imaging. Contrast-enhanced MRI of the brain characterized a large *en plaque* meningioma involving the left middle cranial fossa and the wing of the sphenoid bone, with significant mass effect on the left orbit and compression of the left optic nerve (Figures 1C, 1D). *En plaque* refers to a sheet-like growth pattern along the dura, often associated with adjacent bone thickening or hyperostosis [4]. The case was escalated to a skull base surgical multidisciplinary team (MDT). Given the lesion’s proximity to the optic nerve, radiotherapy was not advised.

Furthermore, a staging CT of the chest, abdomen, and pelvis showed no evidence of metastatic disease. The patient subsequently underwent surgical resection. Histopathological examination confirmed a WHO grade one meningioma [5]. The tumor showed classic meningothelial morphology, arranged in lobules with a whorled architecture. The cells were uniform with occasional intranuclear pseudo-inclusions, and scattered psammoma bodies were present. Mitotic activity was low, with no necrosis or high-grade features. Histopathological images are not included in this report as they were not available to the authors at the time of manuscript preparation; however, the pathological findings were obtained directly from the formal histopathology report issued by the reporting pathologist.

## Discussion

This case underscores the critical need for vigilance in assessing patients presenting with facial pain and sensory disturbances. While TN is typically a clinical diagnosis, atypical features such as persistent pain, sensory deficits, visual symptoms, or neurological signs warrant imaging to exclude secondary causes [3,6]. 

The trigeminal nerve (cranial nerve V) provides sensory innervation to the face, comprising three divisions: ophthalmic (V1), maxillary (V2), and mandibular (V3), with V3 also carrying motor fibers to the muscles of mastication. Due to its extensive anatomical distribution, lesions in regions such as the sphenoid wing or cerebellopontine angle may result in symptoms mimicking TN via compression or irritation of these branches [1,3].

According to NICE Clinical Knowledge Summaries (CKS), TN typically presents with severe, unilateral, electric shock-like pain in the distribution of the trigeminal nerve, lasting seconds to minutes, and provoked by stimuli such as talking, eating, or light touch. The pain is usually episodic with pain-free intervals and without persistent sensory changes [6]. Autonomic features like lacrimation or eyelid edema may occasionally be present [6,7].

However, the patient in this case presented with several red flags. Although the facial pain had some characteristic features of TN, including electric shock-like pain precipitated by talking and chewing, the presentation also included a prolonged three-hour episode of subjective facial numbness, left-sided proptosis, periorbital symptoms, and diplopia on lateral gaze, which raised concern for structural pathology involving the skull base or orbit and prompted further investigation. Furthermore, the nature of the patient’s pain was persistent rather than the short, sharp episodes classically seen in primary TN, with no clear refractory periods. Proptosis and diplopia are not features of classical TN and strongly suggest a local mass effect involving adjacent cranial nerves or orbital structures. Involvement of the ophthalmic (V1) division, particularly in the presence of sensory loss, is atypical for classical TN and should raise suspicion for secondary causes. Primary TN more commonly affects the maxillary (V2) and mandibular (V3) divisions and is typically not associated with objective neurological deficits [7,8].

Meningiomas are typically benign, slow-growing tumors that may present insidiously with symptoms depending on their size and location. Involvement of the sphenoid wing can lead to orbital and cranial nerve compression, as observed in this case [9,10]. Proptosis and diplopia should prompt consideration of orbital or skull base pathology. In this case, proptosis was consistent with orbital involvement, and diplopia on left lateral gaze raised clinical suspicion of left abducens nerve involvement, although objective confirmation of abducens nerve palsy was not formally established at the time of initial presentation. Subsequent ophthalmological assessment confirmed proptosis of approximately 5 mm and diplopia restricted to extreme positions of gaze, with relative preservation of visual acuity and color vision bilaterally. CT scanning with bone windows can reveal characteristic features of meningiomas involving bony structures. However, MRI remains the gold standard for diagnosis and surgical planning [3,10]. Early imaging in this patient significantly altered the diagnostic and management trajectory, potentially preventing further neurological compromise. 

In cases of sphenoid wing meningiomas with optic nerve involvement, factors such as tumor location and proximity to neurovascular structures as well as symptoms help to guide management. When there is optic nerve compression, surgery is generally preferred as it allows for immediate decompression and potential preservation or improvement of visual function. Surgical intervention for skull base located tumors carries risk of cranial nerve injury, vascular complications and incomplete resections due to anatomical limitations. Radiotherapy, including stereotactic radiosurgery, may be considered in selected cases of residual, recurrent, or unresectable disease, offering good tumor control rates. However, its use is limited in lesions located adjacent to the optic apparatus due to the high risk of radiation-induced optic neuropathy [9,10].

From an emergency medicine perspective, this case reinforces several important principles. Firstly, it demonstrates the value of a thorough neurological examination in patients presenting with headache or facial pain to identify red flags and screen for alternative causes. Identifying signs such as cranial nerve involvement or ocular abnormalities should subsequently trigger early investigation [3,6]. Secondly, it highlights the importance of considering secondary causes even in seemingly benign presentations of TN. Although primary TN is a common and often isolated condition, it is important to have a low threshold for ruling out secondary causes such as tumors, particularly when atypical features are present [3,11].

Additionally, this case illustrates the ever-important multidisciplinary approach to care. The coordination between emergency medicine, medical, ophthalmology, radiology, and neurosurgical teams facilitated timely investigation and consideration of an onward definitive management plan. Understanding the pathways for escalation, onward referral, and MDT discussion is vital for ED clinicians managing complex neurological presentations [2,3,6].

Finally, this case highlights the importance of holistic care in the emergency setting. Addressing practical considerations such as driving safety is essential, particularly when a neurological diagnosis may impair function or require DVLA notification [12]. In addition, the emotional impact of discovering a possible brain tumor must also not be underestimated. It was important in this case to make use of the department’s designated private space for sensitive discussions. Providing timely, compassionate communication about the investigation findings and immediate plan moving forward, including the need for further imaging and specialist review, helped to reduce anxiety, build trust, and ultimately ensure the patient felt supported and informed throughout this life-changing, unexpected medical journey [3,13].

Several limitations should be acknowledged. First, the available documentation does not establish whether the patient's transient facial numbness corresponds to an objectively demonstrable trigeminal sensory deficit. Second, the postoperative neurological and visual outcomes, extent of tumor resection, and subsequent imaging follow-up are unavailable. While the anatomical location of the left sphenoid wing meningioma and its relationship to the trigeminal nerve provide strong clinical and radiological support for it being the symptomatic lesion, a definitive causal relationship between this lesion and the patient's trigeminal pain cannot be established with absolute certainty. Although a synchronous right cerebellopontine angle meningioma was identified, it is unclear whether this lesion underwent subsequent surveillance or whether formal assessment for neurofibromatosis type 2-related schwannomatosis or another tumor-predisposition syndrome was undertaken; these limitations restrict interpretation of the long-term outcome and the significance of multiple meningiomas in this patient.

## Conclusions

In the emergency department setting, recognizing atypical features in patients presenting with headache or facial pain is vital, particularly when symptoms may suggest TN but are accompanied by red flags such as persistent sensory disturbance, visual symptoms, proptosis, diplopia, or objective cranial nerve abnormalities. Classical TN does not typically involve objective neurological deficits or ocular signs, and their presence should mandate early neuroimaging and consideration of secondary causes. Emergency clinicians must therefore maintain a broad differential diagnosis and a low threshold for imaging when presenting features are inconsistent with classical TN, as a thorough neurological examination including formal cranial nerve assessment remains essential in identifying subtle signs of intracranial pathology.

This case illustrates how a skull base meningioma may masquerade as primary TN and that meningiomas involving the sphenoid wing represent a diagnostically challenging entity requiring coordinated multidisciplinary management. Although a direct causal relationship between every presenting symptom and the left sphenoid-wing meningioma cannot be established from this single case, the combination of facial sensory symptoms, proptosis, and diplopia prompted investigation for secondary structural pathology. The presence of multiple meningiomas should prompt consideration of an underlying tumor-predisposition syndrome where clinically appropriate. Holistic emergency care must also extend beyond investigation and diagnosis to encompass practical safety considerations and compassionate communication of unexpected findings, both of which are integral to high-quality practice.
